# Multivariable regression analysis of perioperative parameters for a novel pulsed solid-state Thulium: YAG laser with high peak power versus Holmium: YAG laser in prostate enucleation

**DOI:** 10.1007/s00345-025-05756-5

**Published:** 2025-07-26

**Authors:** M. F. von Bargen, M. Glienke, S. Tonyali, A. Sigle, K. Wilhelm, M. Schoenthaler, C. Gratzke, A. Miernik

**Affiliations:** 1 Faculty of Medicine, Department of Urology, University of Freiburg Medical Center, Hugstetter Str. 55, 79106 Freiburg, Germany; 2 Department of Urology, Istanbul University, Istanbul School of Medicine, Istanbul, Turkey

**Keywords:** Benign prostate hyperplasia (BPH), Endoscopic enucleation of the prostate (EEP), Holmium laser enucleation of the prostate (HoLEP), Pulsed thulium laser enucleation of the prostate (pulsed ThuLEP)

## Abstract

**Purpose:**

Due to its physical properties, endoscopic enucleation of the prostate (EEP) with the pulsed solid-state Thulium: YAG laser (pulsed ThuLEP) presents a promising alternative to the widely used Holmium: YAG laser (HoLEP). This study aims to compare perioperative parameters of EEP performed using a novel 100 W pulsed Thulium: YAG laser with high peak power versus a standard 100 W Holmium: YAG laser in patients with benign prostatic hyperplasia (BPH).

**Methods:**

A retrospective analysis was conducted on 312 patients undergoing laser EEP, comprising 80 pulsed ThuLEP and 232 HoLEP procedures. Outcomes were adjusted for key perioperative variables (age, American Society of Anesthesiologists score, hemoglobin, preoperative prostate volume, prostate-specific antigen levels) through multivariable regression analysis. Comparisons between the two techniques utilized adjusted means and marginal contrast analysis.

**Results:**

Baseline characteristics were comparable across groups. Pulsed ThuLEP demonstrated significantly shorter operative times, with a reduction of 6.23 min in total surgery time (*p* = 0.006) and 4.36 min in enucleation time (*p* = 0.001) compared to HoLEP. Although pulsed ThuLEP showed faster enucleation speed, it did not reach statistical significance (*p* = 0.095). Laser energy consumption was comparable (*p* = 0.191). Additionally, pulsed ThuLEP was associated with reduced hospitalization time (4.19 vs. 4.65 days, *p* < 0.001) and lower maximum postoperative pain scores (0.78 vs. 4.23, *p* < 0.001).

**Conclusion:**

The novel pulsed solid-state Thulium: YAG laser offers a viable and effective alternative to the established Holmium: YAG laser for EEP. Advantages of pulsed ThuLEP include shorter operative duration, reduced length of hospital stay, and significantly lower postoperative pain, making it a compelling option for surgical management of BPH.

**Trial registration:**

German Clinical Trials Register number: DRKS00031676.

## Introduction

Benign prostatic hyperplasia (BPH) is a common medical condition affecting aging men. It can lead to lower urinary tract symptoms (LUTS) that significantly affect the quality of life of the patient [1, 2]. In the past, surgical management of BPH included transurethral incision, transurethral resection of the prostate (TURP), or open simple prostatectomy (OP) depending on prostate size. The surgical landscape for the treatment of BPH has evolved significantly over time. Traditional treatments have been supplemented or replaced by more modern approaches like endoscopic enucleation of the prostate (EEP) [3]. Current guidelines now consider EEP as a standard procedure, regardless of the prostate volume [3–5].

With the introduction of the Holmium: YAG laser enucleation of the prostate (HoLEP), the landscape of BPH treatment has advanced significantly. Current guidelines recommend laser enucleation of the prostate (LEP) due to the lower risk of bleeding and shorter hospitalization period compared to TURP [4–7]. Since the debut of HoLEP in 1998 by Fraundorfer and Gilling [8, 9], several new laser sources and techniques have been introduced for the treatment of BPH [10]. The latest addition is the pulsed solid-state Thulium: YAG (Tm: YAG) laser. It has a wavelength of 2.013 μm, which approaches the absorption peak of water at 1.92 μm, and a much higher peak power of 3,700 W compared to other Thulium lasers [11].

It should be noted that the pulsed Tm: YAG laser has a completely different operating mode than a thulium fiber laser, and its characteristics also differ from a continuous Tm: YAG laser [11]. Compared to a pulsed Tm: YAG laser, the thulium fiber laser has longer pulses but with a much lower peak power. Although both the pulsed and continuous Tm: YAG lasers use Tm-doped YAG crystals as active mediums, the continuous energy output of the continuous wave Tm: YAG laser generates more heat [11]. These differences mean that these three laser types are not interchangeable, despite being commonly referred to as thulium lasers.

Because of its physical properties, the pulsed Tm: YAG laser unites the attributes of Holmium: YAG (Ho: YAG) lasers and thulium fiber lasers [12], making it a particularly interesting alternative to the Ho: YAG laser. Compared to Ho: YAG lasers, the pulsed Tm: YAG laser results in lower tissue penetration and less tissue damage [13] as well as a larger cavitation bubble [11] and more efficient ablation [14], thus facilitating EEP [14, 15]. In addition, the pulsed Tm: YAG laser has shown little to no charring/carbonization in porcine kidney models, whereas carbonization was common with both thulium fiber lasers and continuous wave Tm: YAG [13, 16].

This study aimed to compare the perioperative outcomes of EEP with a pulsed Tm: YAG laser to a standard Ho: YAG laser in patients with BPH.

## Materials and methods

This study was performed in line with the principles of the Declaration of Helsinki. The local ethics committee of Albert-Ludwigs-University of Freiburg, Germany, approved the study (Approval number: 22-1465-S1-retro). This retrospective study included 80 patients who underwent pulsed ThuLEP between September 2022 and December 2022 and compared them to 232 patients who underwent HoLEP between January 2022 and August 2022.

Patients were included if they had prostate enlargement causing diagnosed bladder outlet obstruction and did not respond to medical treatment. Both patients with or without refractory urinary retention and/or refractory hematuria were included.

Demographic and clinical data were extracted from patients’ electronic medical records. Baseline characteristics included age, prostate volume (measured by transabdominal ultrasound), prostate-specific antigen (PSA), International Prostate Symptom Score (IPSS), and quality of life questionnaires (QoL), uroflowmetry, post-void residual bladder scan, and American Society of Anesthesiologists (ASA) risk classification.

Antiplatelet therapy with acetylsalicylic acid was not stopped preoperatively, whereas antiplatelet therapy with clopidogrel was paused 7 days prior to surgery. No surgery was performed while the patient was actively treated with anticoagulants except aspirin: Anticoagulation with vitamin K antagonists was bridged while direct oral anticoagulants (rivaroxaban or edoxaban) were paused two days preoperatively.

Perioperative data included duration of surgery (between enucleation start and urinary catheter placement), enucleation time, total laser energy, weight of the enucleated tissue, and speed of enucleation. The duration of catheterization and hospitalization, and peak pain reported on a numeric rating scale (NRS) after surgery completed the perioperative data up to 30 days post-surgery [17].

### Surgical procedure

A standard solid-state Holmium: YAG laser (Ho: YAG Sphinx, LISA Laser Products GmbH, Katlenburg-Lindau, Germany) with a maximum power of 100 W in combination with a laser fiber with a diameter of 550 μm fiber (RigiFib, LISA Laser products OHG, Katlenburg-Lindau, Germany) was used for HoLEP procedures. The laser has a wavelength of 2123 nm. The laser settings were set to 80 W (2.5 J and 32 Hz).

Pulsed ThuLEP was performed with a novel pulsed solid-state Tm: YAG laser which emits laser radiation at a wavelength of 2013 nm with 100 W power (Dornier Thulio^®^ laser, Dornier MedTech Systems GmbH, Wessling, Germany). A 600 μm diameter laser fiber (Dornier Thulio^®^ Performance Fiber, Dornier MedTech Systems GmbH, Wessling, Germany) was used for the procedure; the laser settings were 100 W at 2 J and 50 Hz. Both HoLEP and pulsed ThuLEP procedures used a 26 Fr continuous flow resectoscope (Richard Wolf GmbH, Knittlingen, Germany).

All procedures were performed using the same en-bloc technique (three horse shoe-like incisions) [18] by three experienced surgeons who had each performed more than 250 EEP. The irrigation fluid (saline 0.9%) was located 60 cm above the surgery table. After enucleation, monopolar electrocautery with a high frequency current cautery device (Erbe Elektromedizin GmbH, Tübingen, Germany) and a monopolar roller ball probe (Richard Wolf GmbH, Knittlingen, Germany) was performed to control bleeding. This procedural step was done in both groups for all patients according to our hospital protocol. The Piranha device (Richard Wolf GmbH, Knittlingen, Germany) was used for tissue morcellation. As final step, a standard 20 Fr three-way urinary catheter with continuous bladder irrigation was inserted.

### Statistical analyses

Data collection and statistical analyses were performed using Statistical Package for the Social Sciences (SPSS^®^) version 26.0 (Chicago, IL, USA) and R version 4.4.0 (R Foundation for Statistical Computing, Vienna, Austria).

The outcomes were adjusted with regard to the key covariates age, ASA score, hemoglobin, preoperative prostate volume, and PSA levels using multivariable regression analysis. This adjusted analysis estimates the true underlying effect of the two lasers while controlling for potential effects of the covariates on the outcomes. This provides the same benefit as a matched analysis (i.e., the potential confounders are similar between the two study groups) without the drawback of only including those patients in the analysis where an appropriate match can be found in both study groups. The multivariable regression analysis and adjusted outcomes therefore allow the inclusion of the entire population while removing the influence of potential confounding factors, therefore increasing the statistical power compared to a matched analysis.

The performances of the two lasers were compared using the differences in adjusted means calculated in the multivariable regression. Continuous data were presented using mean and standard deviation (SD) or 95% confidence interval (95% CI). Categorical data were reported using the number of events and percentage. Statistical significance was determined using the Wilcoxon rank sum test for unadjusted continuous data, Fisher’s exact test or Pearson’s Chi-squared test for unadjusted categorical data, and marginal contrast analysis for adjusted data. A *p*-value < 0.05 was considered statistically significant.

## Results

312 patients were included in the study. 80 of these underwent pulsed ThuLEP with the pulsed solid-state Thulium: YAG laser and 232 underwent HoLEP with a solid-state Holmium: YAG laser. The patient groups were comparable regarding preoperative characteristics (Table 1).

**Table 1:** Preoperative patient characteristics

Outcome	HoLEP		Pulsed ThuLEP		
	*N*	Value	*N*	Value	*p*-Value
Age, year, mean (SD)	232	70.97 (8.58)	80	70.55 (7.59)	0.513
ASA, *n* (%)	232		80		0.613
1		18 (7.76%)		6 (7.50%)	
2		121 (52.16%)		48 (60.00%)	
3		91 (39.22%)		26 (32.50%)	
4		2 (0.86%)		0 (0%)	
Hemoglobin, g/dl, mean (SD)	232	14.48 (1.28)	80	14.48 (1.26)	0.912
Preoperative prostate volume, ml, median (IQR)	232	90 (67–120)	79	100 (70–133)	0.072
Prostate-specific antigen, ng/ml, median (IQR)	228	5.29 (2.98–8.13)	79	5.29 (3.45–7.94)	0.789
International Prostate Symptom Score, mean (SD)	128	20.07 (6.62)	43	20.23 (7.02)	0.939
Preoperative quality of life score, median (IQR)	99	4 (3–4)	42	4 (3-4.75)	0.989
Peak flow rate, ml/s, median (IQR)	154	10.6 (7.5–14.0)	49	12.1 (8.0-14.5)	0.375
Preoperative post-void residual volume, ml, median (IQR)	180	70 (30–150)	63	80 (40–200)	0.282

*ASA* American Society of Anesthesiologists risk classification; *HoLEP* Holmium: YAG laser enucleation of the prostate; *SD* standard deviation; *IQR* interquartile range; *pulsed ThuLEP* pulsed Thulium: YAG laser enucleation of the prostate

Both unadjusted and adjusted perioperative parameters are reported in Table 2. In the unadjusted raw data, pulsed ThuLEP was associated with significantly shorter hospital stays than HoLEP (4.21 days [95% CI 4.01–4.40] versus 4.64 days [95% CI 4.48–4.80], *p* < 0.001), a significantly lower maximum pain score (0.78 [95% CI 0.57–0.99] versus 4.25 [95% CI 3.62–4.88], *p* < 0.001), and a higher enucleation speed (4.44 g/min [95% CI 4.04–4.85] versus 3.64 g/min [95% CI 3.28–4.01], *p* < 0.001). The mean enucleated prostate weight was 80.04 g (95% CI 69.11–90.96) for the pulsed ThuLEP group and 69.56 g (95% CI 64.14–74.98) for the HoLEP group (*p* = 0.049). No significant differences were observed in unadjusted outcomes between pulsed ThuLEP and HoLEP with regards to surgery time (*p* = 0.284), enucleation time (*p* = 0.056), total laser energy (*p* = 0.800), and duration of catheterization (*p* = 0.663).

**Table 2:** Perioperative clinical outcomes, unadjusted and adjusted for age, ASA score, hemoglobin, preoperative prostate volume, and PSA

Outcome	*N*	Unadjusted, mean (95% CI)			Adjusted, mean (95% CI)		
		HoLEP	Pulsed ThuLEP	*p*-value	HoLEP	Pulsed ThuLEP	*p*-value
Surgical time, min	274	44.92 (41.80-48.04)	42.50 (37.17–47.83)	0.284	46.00 (43.41–48.60)	39.77 (36.79–42.76)	0.006
Enucleation time, min	295	21.83 (20.28–23.38)	18.86 (16.73–20.99)	0.056	22.20 (20.69–23.70)	17.84 (16.40-19.28)	0.001
Enucleated prostate weight, g	296	69.56 (64.14–74.98)	80.04 (69.11–90.96)	0.049	72.34 (68.92–75.77)	72.26 (66.93–77.58)	0.979
Enucleation speed, g/min	295	3.64 (3.28–4.01)	4.44 (4.04–4.85)	< 0.001	3.71 (3.36–4.07)	4.25 (3.84–4.67)	0.095
Total laser energy, J	293	47998.13 (45184.69–50811.57)	49325.78 (42667.62–55983.94)	0.800	49146.77 (46565.25–51728.29)	46159.65 (42527.72–49791.59)	0.191
Maximum pain score, NRS	306	4.25 (3.62–4.88)	0.78 (0.57–0.99)	< 0.001	4.23 (3.62–4.93)	0.78 (0.59–1.02)	< 0.001
Catheterization time, days	306	2.33 (2.21–2.45)	2.21 (2.08–2.33)	0.663	2.34 (2.22–2.46)	2.18 (2.06–2.31)	0.078
Length of stay, days	306	4.64 (4.48–4.80)	4.21 (4.01–4.40)	< 0.001	4.65 (4.49–4.80)	4.19 (3.99–4.39)	< 0.001

*95% CI* 95% confidence interval; *NRS* numeric rating scale; *HoLEP* Holmium: YAG laser enucleation of the prostate; *pulsed ThuLEP* pulsed Thulium: YAG laser enucleation of the prostate

A multivariable regression analysis was used to adjust the outcomes for potential confounding variables (age, ASA score, hemoglobin, preoperative prostate volume, and PSA levels). In the adjusted data, the surgery time was significantly shorter with pulsed ThuLEP (39.77 min, 95% CI 36.79–42.76) than with HoLEP (46.00 min, 95% CI 43.41–48.60, *p* = 0.006). The enucleation step was also significantly shorter: it was completed within 17.84 min (95% CI 16.40-19.28) with pulsed ThuLEP compared to 22.20 min (95% CI 20.69–23.70) with HoLEP (*p* = 0.001). Although the enucleation speed was numerically higher with pulsed ThuLEP (4.25 g/min, 95% CI 3.84–4.67) than with HoLEP (3.71 g/min, 95% CI 3.36–4.07), the difference did not reach statistical significance after adjustment (*p* = 0.095). The total laser energy was 46.2 kJ (95% CI 42.5–49.8) for pulsed ThuLEP and 49.1 kJ (95% CI 46.6–51.7) for HoLEP (*p* = 0.191). Pulsed ThuLEP was further associated with an 81.6% reduction in peak postoperative pain score (0.78 [95% CI 0.59–1.02] versus 4.23 [95% CI 3.62–4.93], *p* < 0.001), and significantly reduced the hospital stay from 4.65 days (95% CI 3.99–4.39) to 4.19 days (95% CI 4.49–4.80) compared to HoLEP (*p* < 0.001). The catheterization duration was comparable between both lasers (*p* = 0.078), and the weight of the enucleates was also similar (*p* = 0.979).

## Discussion

This retrospective analysis shows that the novel pulsed Tm: YAG laser is at least as effective as the commonly used Ho: YAG laser for EEP, while it increases efficiency by shortening surgery time. Pulsed ThuLEP may even result in a better perioperative recovery indicated by less postoperative pain and a shorter hospital stay.

Both the American Urological Association (AUA) guidelines and the European Association of Urology (EAU) guidelines have already acknowledged laser enucleation as a size-independent, minimally invasive treatment option for BPH [3, 6, 7]. EEP has gained traction over the last decade due to its lower risk of bleeding and shorter hospitalization time compared to traditional TURP [3]. Our study corroborates these findings but adds new insights by comparing the novel pulsed Tm: YAG laser with a 100 W Ho: YAG laser– something previously absent in the literature. It is important to note that both lasers utilized in the study were in pulsed mode, a feature that has been shown to be more conducive for enucleation of prostatic tissue [14, 15].

The results of the regression analysis showed that both surgery time and enucleation time were significantly faster with pulsed ThuLEP than with HoLEP. Pulsed ThuLEP was also found to be more efficient despite our surgeons having more experience with HoLEP, which may have influenced the outcome. We observed similar energy requirements for the pulsed Thulium: YAG laser and the Holmium: YAG laser, which has also been described elsewhere [12].

Interestingly, our study revealed advantages for pulsed ThuLEP in terms of lower maximum postoperative pain. This could possibly be attributed to the pulsed Tm: YAG laser’s higher water absorption, which leads to a reduced tissue penetration compared to the Ho: YAG laser. That might in turn reduce postoperative discomfort.

The only previous publication on pulsed ThuLEP is a single-arm study that found the approach to be safe and effective [19]. They reported mean surgery times of 40.8 min, enucleation times of 18.3 min, and enucleation speed of 4.4 g/min [19], which is very similar to the outcomes reported in this study.

The characteristics of the pulsed Tm: YAG laser show substantial differences to both continuous Tm: YAG lasers and thulium fiber lasers [11]. Therefore, we do not consider the pulsed Tm: YAG laser to be comparable to the other thulium lasers, and think that a direct comparison of our findings to previous literature comparing ThuLEP with either of these other thulium lasers to HoLEP might be misleading.

While our findings are promising, they are not without limitations. The retrospective design inherently imposes some bias. The expertise and experience level of the surgeons with HoLEP might have influenced the results, as might numerous other variables such as slight differences in enucleation technique between the surgeons. Even though we observed a shorter operative time with pulsed ThuLEP, this did not translate into a significant improvement of enucleation speed in the multivariable analysis. Furthermore, the German health insurance system requires a minimum of 2 days of catheterization after pulsed ThuLEP for full reimbursement, even though many patients would have been eligible for catheter removal earlier. In addition, hospital policy allows patients to stay for a minimum of three days after the surgery, and some patients either arrived a day early or were discharged a day later if they had to travel far. This limits the use of length of stay as a meaningful performance metric. Furthermore, any follow-up once the patient left the hospital was done by the patient’s local urologist; therefore, no follow-up data is available beyond the time the patient was discharged from the hospital. Therefore, the subjective pain scores used to quantify postoperative pain in this study could not be correlated with additional pain-related outcomes such as analgesic use as this data was not available once the patient left the hospital. Finally, other covariates that were not considered in this analysis (such as the learning curve) may be present and influence the results. The findings reported here should be confirmed in a future randomized controlled trial.

## Conclusion

In conclusion, our study adds to the growing body of evidence suggesting that pulsed solid-state Thulium: YAG lasers offer a viable alternative to Holmium: YAG lasers for EEP. Both types of lasers exhibited comparable efficacy profiles, but in a multivariable regression analysis adjusting for key covariates, the pulsed solid-state Thulium: YAG laser showed advantages in terms of surgery time and postoperative comfort. As we move forward in this rapidly evolving field, further research is required to ascertain the long-term outcomes and to explore the influence of various factors like surgeon experience and specific laser settings on the efficacy and safety of EEP.

## Data Availability

The data set is with the corresponding author.
